# Professional identity and work-related quality of life among obstetric nurses: Mediating role of psychological resilience and moderating effect of internet+ participation willingness

**DOI:** 10.1371/journal.pone.0338187

**Published:** 2025-12-12

**Authors:** Lingling Wu, Weiwei Cai, Fei Wang, Yazhuo Wang, Chang Wang

**Affiliations:** 1 School of Nursing, Bengbu Medical University, Bengbu, Anhui Province; 2 Department of Nursing, Bengbu Third People’s Hospital, Bengbu, Anhui Province; 3 The Second Affiliated Hospital of Anhui Medical University, Hefei, Anhui, China; Alexandria University Faculty of Nursing, EGYPT

## Abstract

**Background:**

As the “Internet plus” era unfolds, the domain of obstetric nursing has ushered in the innovative development model of Internet plus Nursing Services(IPNS). This shift has led to alterations in the professional environment and work demands faced by obstetric nursing personnel, consequently bringing the work-related quality of life of these nursing professionals to the forefront of attention.

**Objective:**

This research aims to investigate the mediating and moderating effects of psychological resilience and the willingness to engage in IPNS among obstetric nurses on the relationship between professional identity and work-related quality of life, under the premise that these factors have a significant impact on work-related quality of life.

**Methods:**

This study selected obstetric nurses from 33 tertiary hospitals in Anhui Province, China, that have implemented IPNS to date and met the specified criteria for inclusion and exclusion. The assessment focused on their professional identity, psychological resilience, work-related quality of life, and willingness to engage in providing “Internet plus Nursing Services.” AMOS was used for mediation effect analysis, and the PROCESS macro in SPSS was employed to investigate moderated mediation effects.

**Results:**

In the context of IPNS, psychological resilience acts as a mediator in the relationship between professional identity and work-related quality of life among obstetric nurses. Furthermore, psychological resilience and the willingness to participate exhibit a moderated mediation effect in this relationship—specifically, psychological resilience modulates the latter segment of the pathway from professional identity to work-related quality of life via the mediation of participation willingness.

**Conclusion:**

The findings suggest that nursing administrators, while focusing on the work-related quality of life of obstetric nurses, should concurrently consider nurses’ psychological resilience and their willingness to participate. This approach facilitates the development of effective employee motivation and support strategies, which in turn can enhance the work-related quality of life of obstetric nurses in the IPNS context.

## Introduction

Against the backdrop of Internet plus Nursing Services(IPNS), internet technology has progressively permeated numerous sectors, including the domain of nursing, which has adopted the innovative model of IPNS [1]. IPNS constitutes a vital component of China’s healthcare service reform. Its implementation not only constitutes a concrete measure in response to the National Nursing Development Plan (2021–2025) to broaden nursing service domains and facilitate the decentralisation of high-quality nursing resources, but also aligns closely with the objective of optimising the healthcare service system outlined in China’s ‘Healthy China 2030’ strategy. It has emerged as a significant pathway to alleviate shortages of primary-level nursing resources and enhance the accessibility of maternal and child healthcare services [2]. In 2023, the National Health Commission and the National Administration of Traditional Chinese Medicine jointly issued the Action Plan for Further Improving Nursing Services (2023–2025), this plan emphasizes that healthcare institutions should strengthen information technology support, actively innovate nursing service models, and achieve continuity of care within hospitals. It calls for a gradual increase in the number of IPNS-enabled healthcare institutions and home-visit nursing service offerings [3]. Through remote nursing consultations and other means, it aims to support primary healthcare facilities within medical consortiums, facilitate the decentralisation of nursing resources, and promote balanced regional development of medical resources. This strategy not only alleviates the uneven distribution of healthcare resources but also enhances patient experience and nursing staff efficiency.

Multiple countries have explored similar digital nursing services. For instance, the United States employs telecommunications technologies like Remote Patient Monitoring (RPM) to deliver remote specialist care, particularly addressing follow-up management for pregnant women with chronic hypertension in rural areas. This enables small rural hospitals to sustain and advance remote clinical healthcare within manageable cost parameters [4]. The United Kingdom, meanwhile, has leveraged the National Health Service (NHS) to establish a national-level digital primary care system, incorporating emergency care data sharing capabilities to improve patients’ emergency treatment experiences [5]. These models have seen widespread adoption across diverse nations and regions, illustrating global trends and innovative practices within the IPNS domain.

By utilizing digital health platforms, this model extends the scope of nursing interventions from institutional healthcare facilities to community-based and home care environments, thereby enhancing the accessibility and efficacy of nursing services for patients [6,7]. As nursing models evolve, the professional environment and work demands faced by obstetric nurses have undergone changes, thereby affecting their work-related quality of life. Prior research has indicated that the factors influencing the work-related quality of life of nursing staff in the context of IPNS encompass professional identity and psychological resilience and are additionally correlated with the degree of participation willingness [8]. Nevertheless, the mechanism by which professional identity influences work-related quality of life, as well as the potential moderating effects of participation willingness and psychological resilience in the context of IPNS, warrant further exploration. Consequently, the present study endeavored to investigate how professional identity affects work-related quality of life among obstetric nurses within the IPNS framework, aiming to enhance the professional development and mental well-being of nursing staff and to offer strategies for elevating the work-related quality of life of nursing professionals.

### Professional identity and work-related quality of life

Professional identity is defined as an individual’s acknowledgment and acceptance of their professional role, with research suggesting the concept has a substantial influence on the work attitude, behavior, and career progression of nurses [9]. Work-related quality of life highlights the physiological and psychological sensations experienced in the workplace, reflecting the quality of the relationship between nurses and their overall working environment [10,11]. Studies suggest that professional identity can positively forecast work-related quality of life; in healthcare, as the level of professional identity among nurses rises, their work-related quality of life correspondingly increases [12]. Individuals with a strong sense of professional identity are more prone to view their work as a crucial component of their self-identity. This perspective leads to greater fulfillment in their work life, increased work engagement, and higher job satisfaction, which in turn elevates their overall work quality of life [13,14]. In the context of IPNS, obstetric nursing services have become increasingly convenient and efficient. However, obstetric nursing demands a high level of professional skills, emergency response capabilities, and psychological resilience from nurses. Obstetric nurses who possess a strong sense of professional identity, upon recognizing the value of their profession and experiencing a sense of well-being, are more likely to be actively involved in clinical work [15].

### The mediating role of psychological resilience

Psychological resilience is the process by which an individual, through the dynamic interaction of abilities and traits, quickly recovers and successfully copes with significant stress and danger [16]. Research indicates that psychological resilience is positively correlated with work-related quality of life among nurses, and the level of psychological resilience significantly affects their work engagement [17]. In the context of IPNS, obstetric nurses bear the critical responsibility of ensuring the life safety of mothers and infants, facing heavy workloads and high risks. These nurses must have not only solid medical knowledge but also a strong sense of responsibility and compassion to safeguard the health and safety of mothers and infants, which can easily lead to job burnout. This, in turn, affects their professional identity and ultimately impacts their work-related quality of life [18]. Consequently, the present study hypothesizes that psychological resilience will serve as a mediator between professional identity and work-related quality of life among obstetric nurses in the IPNS context.

### The moderating role of willingness to participate

Obstetric nursing work is distinct from nursing in other departments and possesses specific characteristics. First, the primary service recipients in obstetrics are healthy mothers and newborns, whose needs for nursing care differ from those of other patients; second, IPNS has broadened the settings for health education beyond the confines of hospitals. Moreover, receiving a high-quality health education in obstetrics requires nurses to possess strong communication and time-management skills, thereby setting higher standards for their nursing capabilities. This also results in differing degrees of willingness among obstetric nurses to engage in IPNS [19]. Current research suggests that the work willingness of healthcare professionals is contingent upon employee resilience, including the capacity to harness psychological, social, cultural, and physical resources to sustain health, thereby influencing their resolve to persevere in work despite discomfort and suffering [20]. Consequently, it can be posited that the participation willingness of obstetric nurses might serve to moderate psychological resilience, which in turn impacts their work-related quality of life. We therefore hypothesize that the willingness to participate in IPNS and psychological resilience among obstetric nurses act as moderated mediators in the influence of professional identity on work-related quality of life.

### Hypothetical research model

**Fig 1:**
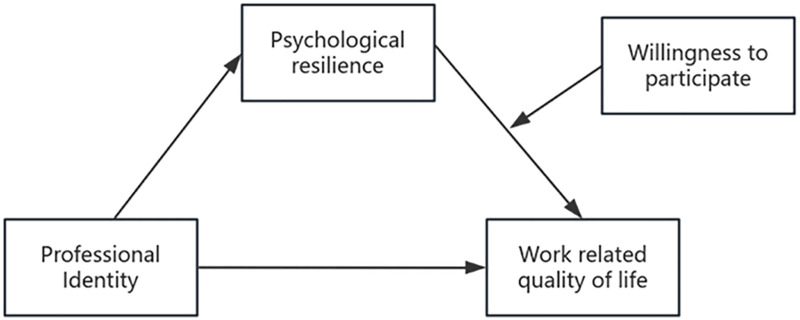
The hypothesized moderated mediation model.

Research on the impact pathways of work-related quality of life among obstetric nurses in the IPNS context is relatively scarce. Hence, this study investigates its underlying mechanism, considering psychological resilience as the mediator and participation willingness as the moderator. Specifically, this study aims to test the following hypotheses (Fig 1):

- Hypothesis 1 (H1): Psychological resilience mediates the effect of professional identity on work-related quality of life.
- Hypothesis 2 (H2): The willingness to participate in IPNS and psychological resilience exhibit a moderated mediating effect in the influence of professional identity on work-related quality of life.

## Methods

### Study design

This study is a cross-sectional observational investigation. We employed convenience sampling to select obstetric nurses from 33 tertiary hospitals in Anhui Province who had implemented IPNS to date and met the inclusion and exclusion criteria specified in the research protocol.

### Sample size

The sample size calculation was performed using 5–10 times the number of study variables. This research selected the Work-related Quality of Life Scale comprising 33 variables. Taking into account a potential 20% non-response rate and the requirement for an expanded sample size, the final sample size was determined to be at least 396 participants.

### Eligibility criteria

We via hospital nursing departments and head nurses, and all those who met the inclusion criteria were invited to participate. Eligible participants included obstetric nurses: ① holding a “Nurse Practice Certificate” and currently registered on duty; ② with at least 1 year of nursing work experience; ③ Researchers or project leaders explain the content of the consent form to participants in a clear and understandable manner. This process ensures that participants are fully informed about the nature and implications of their involvement, and it affirms that those who provided informed consent did so voluntarily and participated in the study willingly. Nurses were excluded if they either ① were not on duty during the study period, or ② were visiting, retired, or rehired. Ultimately, 522 valid questionnaires were obtained.

### Ethical considerations

This study has been approved by the Ethics Committee of the Third People’s Hospital of Bengbu City, Anhui Province (Ethics Approval No. K40 [2023]). All research participants have provided written informed consent. This research was conducted in accordance with the Declaration of Helsinki. Recruitment for this study commenced on 1 September 2023 and concluded on 1 February 2024.

### Reporting format

We adhered to the strengthening the reporting of observational studies in epidemiology (STROBE) guidelines for reporting observational studies [21].

### Research

#### General.

The survey included variables such as birth sex gender, age, highest educational attainment, marital status, years of work experience, professional title, rank, average monthly income, total duration of obstetric-related professional training, years of clinical teaching experience, willingness to engage in IPNS, and whether home nursing services were provided to pregnant and postpartum women, among others.

#### Nurse professional identity assessment scale.

The Nurse Professional Identity Assessment Scale, developed by Liu [22], was used in this study. This scale includes 30 items covering five dimensions: professional cognitive evaluation, professional social skills, professional social support, professional frustration coping, and professional self-reflection. Five response options are available for each item (strongly disagree, disagree, sometimes agree, agree, and strongly agree), which are scored from 15 points, respectively, yielding a total score range of 30150 points. The scale exhibits robust psychometric properties, with a Cronbach’s alpha coefficient of 0.938, reflecting satisfactory reliability and validity.

#### Nurse psychological resilience scale.

The Conner-Davidson Resilience Scale, revised by Zhang [23], was also employed in this study. This scale covers three dimensions: tenacity (12 items), strength (7 items), and optimism (4 items), with a total of 25 items. The scale demonstrates good psychometric properties, with a Cronbach’s alpha coefficient of 0.91, reflecting satisfactory reliability and validity.

#### Work-related quality of life scale.

Finally, the Work-related Quality of Life Scale translated and revised by Shao et al. [24,25], was used in this study. This scale consists of 33 items covering seven dimensions: work conditions, work stress, work control, work-family balance, work evaluation, general well-being, and career satisfaction. A 5-point rating scale is employed, with scores ranging from 1 (strongly disagree) to 5 (strongly agree) points, yielding a total score range of 33165 points. The scale exhibits robust psychometric properties, with an overall Cronbach’s alpha coefficient of 0.939, reflecting satisfactory reliability and validity.

#### Covariates.

In this study, we controlled for variables that could potentially bias the results, as informed by prior research and correlation analyses. The controlled variables encompassed demographic factors and relevant covariates-specifically, rank and the total duration of involvement in obstetric-related professional training.

#### Survey method.

Electronic questionnaires were disseminated through the online Questionnaire Star platform (Changsha Ranxing Information Technology Co., Ltd., Changsha, Hunan, China). Following communication with and approval from hospital administrators, the questionnaires were distributed by obstetric department managers via WeChat groups. All items were designated as “required” to ensure the completeness and validity of the responses, and each IP address was limited to a single submission. With adherence to the specified inclusion and exclusion criteria, 522 questionnaires were distributed, yielding 522 valid returns, for a 100% effective recovery rate.

#### Statistics.

Data entry and organization were performed using Microsoft Excel (Microsoft Corp., Redmond, WA, USA), and statistical analyses were carried out with SPSS 26.0 (IBM Corp., Armonk, NY, USA). Measurement data conforming to a normal distribution were expressed as mean ± standard deviation values, and categorical data were described using frequencies (%). Mediation effect models were constructed using AMOS (IBM Corp., Armonk, NY, USA), and the PROCESS macro in SPSS, in conjunction with the bootstrap method, was employed for testing moderation effects, with model 14 selected for hypothesis testing.

## Results

### Common method bias test

As mentioned, this study used the Questionnaire Star platform for data collection, which could potentially introduce common method bias. Consequently, Harman’s single-factor test was employed to assess common method bias [26]. The results indicated that the first factor accounted for 45.083% of the total variance (<50%), suggesting that common method bias was not a significant concern.

### Descriptive statistics

The general information of the 522 obstetric nurses involved in this study, the variable assignment methodology, and the work-related quality of life scores for nurses with differing characteristics are detailed in Tables 1 and 2. Table 3 displays the scores from the Nurse Professional Identity Assessment Scale, the Psychological Resilience Scale, and the Work-related Quality of Life Scale for obstetric nurses.

**Table 1:** Assignment Methods for Independent Variables.

Independent variable	Assignment method
Level	N1 = 1; N2 = 2; N3 = 3; N4 = 4; N5 = 5
Total duration of obstetrics-related training(months)	0 ~ 1 = 1;1 ~ 3 = 2;3 ~ 6 = 3; ＞ 6 = 4
Willingness to participate	Strongly willing = 1; Quite willing = 2; General = 3; Unwilling = 4; Strongly unwilling = 5
Professional identity scale	Original value input
Psychological resilience scale	Original value input

**Table 2:** Comparison of Work-Related Quality of Life Scores Among 522 Obstetric Nurses with Different Characteristics (Scores, *x̅* ± s).

Variable	Classification	N (%)	*x* ®± s	*t/F*	*P*
Sex	Male	1 (0.2)	99.00 ± 0.00	0.881	0.379
	Female	521 (99.8)	121.04 ± 24.99		
Age (years)	≤25	25 (4.8)	114.64 ± 21.65	0.854	0.465
	≥25, ≤ 35	327 (62.6)	121.45 ± 26.31		
	≥35, ≤ 45	120 (23.0)	119.92 ± 23.11		
	≥45	50 (9.6)	122.18 ± 20.91		
Highest education	Under college	1 (0.2)	130.00 ± 0.00	1.630	0.182
	College degree	49 (9.4)	113.59 ± 21.22		
	Bachelor’s degree	466 (89.3)	121.73 ± 25.27		
	Graduate degree	6 (1.1)	123.00 ± 26.87		
Marital status	Unmarried	98 (18.8)	118.93 ± 25.15	0.472	0.624
	Married	419 (80.3)	121.52 ± 24.99		
	Divorce	5 (0.9)	117.60 ± 23.38		
Years of service (years)	5-10	287 (55.0)	121.16 ± 25.83	0.529	0.663
	10-15	117 (22.4)	119.58 ± 25.72		
	16-20	43 (8.2)	118.93 ± 21.74		
	≥20	75 (14.4)	123.75 ± 22.33		
Title	Nurse	37 (7.1)	116.89 ± 28.10	1.262	0.284
	Senior nurse	204 (39.1)	123.35 ± 25.56		
	Nurse in charge	261 (50.0)	119.30 ± 24.52		
	Deputy chief nurse	15 (2.8)	126.93 ± 16.44		
	Chief nurse	5 (1.0)	125.80 ± 15.43		
Level	N1	50 (9.6)	113.42 ± 25.21	3.395	0.009
	N2	94 (18.0)	128.06 ± 25.66		
	N3	202 (38.7)	120.22 ± 25.81		
	N4	161 (30.8)	119.72 ± 23.44		
	N5	15 (2.9)	126.00 ± 14.19		
Monthly income (RMB yuan)	≤3000	21 (4.0)	119.81 ± 33.29	0.981	0.417
	3000-5000	140 (26.8)	119.70 ± 27.56		
	5000-7000	139 (26.7)	118.93 ± 24.96		
	7000-9000	116 (22.2)	124.65 ± 22.64		
	≥9000	106 (20.3)	121.65 ± 21.85		
Total duration of obstetrics-related training (months)	0-1	48 (9.2)	115.69 ± 25.77	3.948	0.008
	1-3	75 (14.4)	116.48 ± 24.75		
	3-6	56 (10.7)	115.14 ± 24.45		
	≥6	343 (65.7)	123.68 ± 24.99		
Years of clinical teaching (years)	0	135 (25.8)	118.15 ± 25.81	0.829	0.478
	1-5	147 (28.2)	121.59 ± 27.23		
	5-10	134 (25.7)	122.60 ± 23.86		
	≥10	106 (20.3)	121.75 ± 21.93		
Willingness to participate	Strongly willing	256 (49.0)	128.69 ± 26.12	17.416	≤0.001
	Quite willing	129 (24.8)	119.02 ± 19.46		
	General	126 (24.1)	109.35 ± 21.88		
	Unwilling	6 (1.1)	103.00 ± 20.39		
	Strongly unwilling	5 (1.0)	93.00 ± 23.82		

**Table 3:** Scores on Professional Identity, Psychological Resilience, and Work-Related Quality of Life Scales Among Obstetric Nurses (N = 522).

Scales and dimensions	Number of entries	Score range (points, *x̅* ± s)	Score (points, *x̅* ± s)
Professional identity scale	30	30-150	102.14 ± 22.398
Occupational cognition evaluation	9	9-45	31.17 ± 6.680
Professional social skills	6	6-30	19.95 ± 4.797
Professional social support	6	6-30	20.26 ± 5.127
Coping with career setbacks	6	6-30	20.78 ± 4.549
Professional self-reflection	3	3-15	9.98 ± 2.622
Psychological resilience scale	23	23-115	81.61 ± 12.482
Tenacity	12	12-60	42.73 ± 6.901
Strength	7	7-35	24.32 ± 4.218
Optimism	4	4-20	14.56 ± 2.349
WRQoL	33	33-165	107.84 ± 24.640
WCS	6	6-30	20.38 ± 5.141
SAW	5	5-25	14.61 ± 4.342
CAW	5	5-25	17.20 ± 3.579
HWI	2	2-10	6.59 ± 1.809
EEN	5	5-25	16.62 ± 4.554
GWB	5	5-25	15.94 ± 5.299
JCS	5	5-25	16.51 ± 4.625

*Note.* CAW, control at work; EEN, employee evaluation; GWB, general well-being; HWI, home-work interface; JCS, job and career satisfaction; SAW, stress at work; WCS, working conditions; WRQoL, Work-related Quality of Life Scale.

### Correlation analysis among main study variables

The correlations among professional identity, psychological resilience, work-related quality of life, and the willingness to participate in IPNS among obstetric nurses are presented in Table 4. According to our data, professional identity and psychological resilience are significantly associated with work-related quality of life. Separately, the willingness to participate shows a small, non-significant negative correlation with work-related quality of life, whereas both professional identity and psychological resilience exhibit positive correlations with work-related quality of life (r = 0.698, *P* < 0.001; r = 0.628, *P* < 0.001).

**Table 4:** Pearson Correlations Among Main Study Variables (r Values, N = 522).

Variable	M	SD	1	2	3	4
1. Professional identity	102.14	22.398	1			
2. Work-related quality of life	107.84	24.640	.698**	1		
3. Psychological resilience	81.61	12.482	.588**	.628**	1	
4. Willingness to participate	1.80	0.909	−.092*	−.085*	−.162**	1

*Note.* * *P* < 0.05, significant at the 0.05 level (two-tailed); ** *P* < 0.001, significant at the 0.01 level (two-tailed). M, mean; SD, standard deviation.

### The mediating role of psychological resilience

This study used AMOS to construct a mediation effect testing model, with the results presented in Tables 5 and 6. The results indicated that the professional identity of obstetric nurses positively predicts psychological resilience (β = 0.71, *P* < 0.001), suggesting that nurses with a stronger sense of professional identity exhibit higher levels of psychological resilience. In other words, obstetric nurses with a greater sense of identification with their nursing work are better equipped to handle work stress and challenges in the context of IPNS. Furthermore, psychological resilience positively predicts work-related quality of life (β = 0.53, *P* < 0.001), implying that nurses with greater psychological resilience enjoy a superior work-related quality of life. The results further suggest that the influence of professional identity on work-related quality of life mediated by psychological resilience among obstetric nurses is significant (β = 0.37, *P* < 0.001), accounting for 50.9% of the total effect. The χ²/df ratio is generally considered to indicate good model fit when below 3. Although this value is slightly elevated, it remains within an acceptable range. The fit indices demonstrate that the model exhibits satisfactory fit (CFI = 0.976, TLI = 0.971, RMSEA = 0.079, SRMR = 0.032). These results indicate that the hypothetical model aligns with the empirical data, and the model fit is acceptable, thereby supporting H2.

**Table 5:** The Mediating Role of Psychological Resilience Between Professional Identity and Work-Related Quality of Life.

Outcome	Model path	β	Estimate	95% CI	
				Lower	Upper
Work-related quality of life	Total effect	0.74	1.367	1.212	1.521
	Direct effect	0.36	0.671	0.522	0.819
	Indirect effect	0.37	0.696	0.690	0.701
	Professional identity → Psychological resilience	0.71	1.511	1.328	1.693
	Psychological resilience → Work-related quality of life	0.53	0.461	0.382	0.539

**Table 6:** Fitness indexes of the model.

	χ²	df	χ²/df	CFI	TLI	RMSEA	SRMR
Standard	–	–	＜3	＞0.9	＞0.9	＜0.08	＜0.08
Actual value	367.718	86	4.276	0.976	0.971	0.079	0.032

### The moderating role of willingness to participate

This study employed the PROCESS plugin in SPSS, using the bootstrap method proposed by Hayes [27] to assess the moderated mediation effect. Model 14 was selected, with a sample size of 5000, to examine the direct path, the initial segment, and the latter segment of the mediated path within a 95% confidence interval (CI). The analysis indicated that the willingness to participate did not exert a moderating effect in the initial segment of the mediated path but did so in the latter segment -namely, the “psychological resilience → work-related quality of life” path. The willingness to participate significantly moderated the process whereby professional identity influenced work-related quality of life via psychological resilience (*P* = 0.0098). First, the effect of professional identity on psychological resilience was significant (β = 0.327, t = 16.589), and the effect of psychological resilience on work-related quality of life was also significant (β = 0.645, t = 8.996), thereby establishing the path through which professional identity influences work-related quality of life via psychological resilience. Second, the interaction item of psychological resilience and willingness to participate significantly affected work-related quality of life (β = 0.164, t = 2.591), indicating that the willingness to participate moderated the relationship between psychological resilience and work-related quality of life, thereby supporting H2. The detailed results are presented in Tables 7 and 8.

**Table 7:** Analysis of the Moderating Role of Willingness to Participate in the Impact of Psychological Resilience on Work-Related Quality of Life.

Index	coeff	SE	t	P	95% CI	
					LLCL	ULCL
Constant	51.5576	4.0948	12.5909	.0000	43.5131	59.6022
Professional identity	.5480	.0395	13.8732	.0000	.4704	.6256
Psychological resilience	.6455	.0717	8.9964	.0000	.5045	.7864
Willingness to participate	.0325	.8113	.0400	.9681	−1.6263	−1.5614
Int.	−.1649	.0636	−2.5916	.0098	−.2899	−.0399

*Note.* CI, confidence interval; LLCL, lower-level confidence interval; ULCL, upper-level confidence interval; SE, standard error.

**Table 8:** Regression Analysis of the Moderated Mediation Model.

	Psychological Resilience			Work-related Quality of Life		
	β	SE	t	β	SE	t
Constant	−33.486	2.066	−16.205	51.557	4.0948	12.590
Professional identity	.327	.019	16.589	.548	.039	13.873
Willingness to participate	–	–	–	.032	.811	.04
Psychological resilience*Willingness to participate	–	–	–	−.164	.063	−2.591
Psychological resilience	–	–	–	.645	.071	8.996
R^2^	.346			.564		
F	275.219			167.705		

*Note.* SE, standard error.

**Fig 2:**
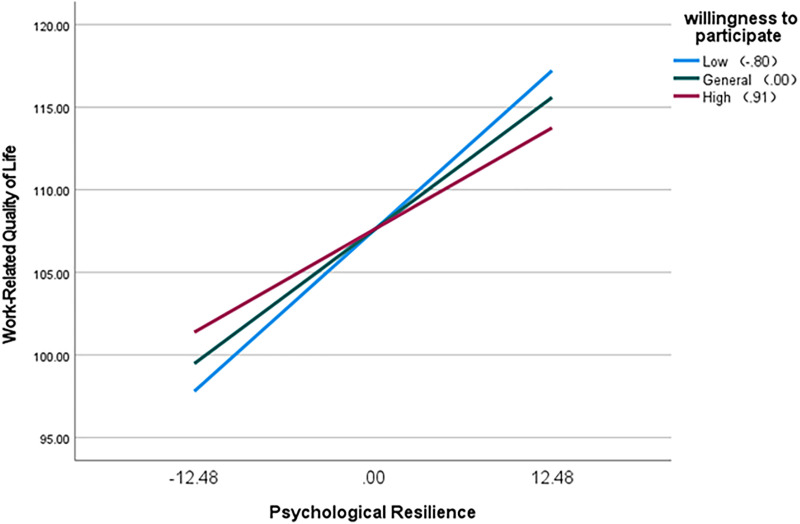
The moderating role of willingness to participate between psychological resilience and work-related quality of life. *Note*. At low, medium and high levels of willingness to participate, the simple slope of psychological resilience on work-related quality of life decreased in turn (0.778 → 0.646 → 0.496), reflecting the negative moderating effect of willingness.

At different levels of psychological resilience, the moderating effect of participation willingness on work-related quality of life varies. When psychological resilience is high (M + 1SD), the negative moderating effect of participation willingness is more pronounced, and the positive moderating effect of psychological resilience on work-related quality of life is more significantly suppressed. When psychological resilience declines to a lower level (M-1SD), the negative moderating effect of participation willingness diminishes significantly, while the positive moderating effect of psychological resilience on work-related quality of life becomes more pronounced (Effect = 0.778, 95% CI = [0.612, 0.943]). Detailed results are presented in Table 9. Further analysis through simple slope tests revealed that, with an increase in psychological resilience among obstetric nurses, their work-related quality of life correspondingly improved. Different levels of participation willingness are depicted by lines of varying colors (Fig 2), and it was noted that the willingness of obstetric nurses to engage in IPNS inhibited the positive effect of psychological resilience on work-related quality of life.

**Table 9:** The Moderating Effect of Willingness to Participate.

Mediating variable		Willingness to participate	Effect	SE	95% CI	
					LLCL	ULCL
Psychological resilience	M −1SD	−.8027	.7778	.0844	.6120	.9437
Psychological resilience	M	.0000	.6455	.0717	.5045	.7864
Psychological resilience	M + 1SD	.9092	.4955	.0960	.3070	.6841

*Note*. CI, confidence interval; LLCL, lower-level confidence interval; ULCL, upper-level confidence interval; M, mean; SD, standard deviation; SE, standard error.

## Discussion

We have focused on the work-related quality of life of obstetric nurses in the context of “Internet plus” and elucidated the mediating and moderating relationships between professional identity, psychological resilience, the willingness to participate in IPNS, and work-related quality of life using a moderated mediation effect model. The findings of this study indicate that, in the “Internet plus” context, the professional identity of obstetric nurses significantly impacts work-related quality of life, exerting both direct and indirect effects via psychological resilience as a mediating variable. Furthermore, the willingness to participate moderates the path from psychological resilience to work-related quality of life.

### Occupational identity and psychological resilience are correlated with work-related quality of life

The positive correlation between professional identity and work-related quality of life identified in this study corroborates prior research by Zhu et al. [28] among nurses in tertiary healthcare institutions. This reinforces the predictive validity of professional identity as a determinant of nurses’ occupational well-being across conventional and innovative nursing service models. Additionally, Wen et al. [29] demonstrated that psychological resilience facilitates stress adaptation, emotional regulation, and optimal work engagement among fever clinic nurses, thereby enhancing their work-related quality of life. These findings align with the present results, confirming the universal role of psychological resilience in bolstering nurses’ psychological well-being and occupational satisfaction. The increased demands on maternity nurses within the IPNS framework—who must concurrently ensure maternal and neonatal safety, address maternal emotional needs, and manage online health guidance and home visits—exacerbate occupational stressors, predisposing staff to burnout. Professional identity functions as an intrinsic motivator for maintaining work engagement, while psychological resilience serves as a critical buffer against occupational stress. Deficiencies in either domain adversely affect nurses’ work-related quality of life [30–32]. Consequently, nursing leadership should emphasize mental health promotion, encourage proactive management of obstetric emergencies, and foster professional identity development. The integration of mindfulness-based interventions, such as meditation, yoga, and breathing exercises may mitigate burnout and stress-related impacts. Collaborative efforts to advance IPNS are essential for sustainable development in this context [33].

### The mediating role of psychological resilience between professional identity and work-related quality of life

The results of this investigation demonstrate that psychological resilience functions as a mediating factor in the association between professional identity and occupational quality of life among obstetric nursing professionals within the framework of ‘Internet Plus nursing services,’ thereby corroborating Hypothesis 1. The mediating influence of psychological resilience aligns with prior research by Yang et al. [34], which identified that individuals exhibiting high levels of psychological resilience demonstrate advantageous psychological attributes such as resilience, optimism, and increased subjective well-being, alongside strengths in personal competencies, familial connections, and social support networks. In this context, occupational identity among obstetric nurses directly impacts their work-related quality of life and exerts an indirect effect through psychological resilience, with the mediating proportion accounting for 50.9% of the total effect. This phenomenon may be attributed to the ‘Internet Plus nursing services’ environment, where elevated occupational identity prompts nurses to perceive their roles as avenues for self-actualization, thereby requiring enhanced psychological resilience to address the complexities of remote care delivery. Excessive empathy fatigue among nurses diminishes their professional engagement. Within the IPNS paradigm, home visitation by obstetric nurses necessitates advanced clinical competencies and communication skills. Nurses with limited professional identity due to inexperience should undergo targeted psychological theory and skills training [35] Those experiencing reduced work engagement stemming from emotional exhaustion or communication barriers should receive supportive interventions. Cultivating a positive clinical environment facilitates maternal nurses in effectively managing negative emotional states, bolstering psychological resilience, adapting to work demands, and improving service quality.

### The moderating role of willingness to participate

The results of this investigation demonstrate that obstetric nurses’ willingness to engage in Internet-based nursing services moderates the latter segment of the pathway whereby professional identity influences work-related quality of life through psychological resilience, thereby corroborating Hypothesis 2. This indicates that the mediating effect is negatively moderated by participation willingness. Specifically, when nurses exhibit lower levels of participation willingness, psychological resilience exerts a more substantial positive impact on work-related quality of life, whereas this influence diminishes at higher levels of willingness. These findings are consistent with prior research by Orrell et al. [36], which indicates that despite high levels of work engagement, perceived meaningfulness, and job satisfaction, demanding and stressful work conditions continue to adversely affect mental health [37]. This suggests that highly motivated nurses may allocate greater effort to their work, thereby partially mitigating the moderating influence of psychological resilience on work-related quality of life. Such observations align with Self-Determination Theory, which posits that intrinsic motivation is intrinsically linked to the fulfillment of three basic psychological needs: autonomy, competence, and relatedness [38]. Consequently, nursing management should consider strategies to address psychological resilience depletion among highly motivated staff. Interventions such as cognitive-behavioral therapy may be employed to enhance occupational well-being, or nurse-led psychological capital initiatives may be implemented to balance resilience and work engagement. Cultivating an obstetric nursing environment that promotes autonomy, reinforces professional identity, and fosters social support addresses fundamental psychological needs, thereby strengthening resilience and improving work-related quality of life [39,40].

## Limitation

A limitation of this study is that the participants were predominantly female and held relatively high professional titles, which may introduce bias. Future research could investigate the impact pathways and mechanisms of work-related quality of life among male or low-level nursing staff in the IPNS context, providing additional information. In addition, this investigation employs a cross-sectional design, thereby precluding causal inferences among variables. Future longitudinal studies are recommended to examine the temporal dynamics of professional identity, psychological resilience, and work-related quality of life. The data collection relies on self-reported measures from nursing professionals, which may introduce response bias. Finally, the sample is restricted to nurses from Anhui Province, potentially limiting the generalizability of the findings across broader populations.

## Conclusion

This study investigated the mediating and moderating roles of professional identity, psychological resilience, willingness to participate, and work-related quality of life among obstetric nurses in the context of IPNS. IPNS effectively mitigates a hospital’s inpatient load via online health guidance and home care services provided by obstetric nurses, promoting the rational distribution of resources and alleviating the burden on clinical staff. Nevertheless, obstetric nurses continue to experience high levels of work stress in this context. Our findings suggest that it is crucial to attend to the psychological resilience of nursing staff and participation willingness, which can subsequently improve work-related quality of life in obstetrics.

## Abbreviations

CAW: control at work
EEN: employee evaluation
GWB: general well-being
HWI: home-work interface
JCS: job and career satisfaction
SAW: stress at work
WCS: working conditions
WRQoL: Work-related Quality of Life Scale
SD: standard deviation
CI: confidence interval
LLCL: lower-level confidence interval
ULCL: upper-level confidence interval
SE: standard error.
